# NFS1 Plays a Critical Role in Regulating Ferroptosis Homeostasis

**DOI:** 10.3390/biom16010032

**Published:** 2025-12-24

**Authors:** Siying Sun, Hanwen Cao, Xuemei Li, Hongfei Liao

**Affiliations:** 1 School of Optometry, Jiangxi Medical College, Nanchang University, Nanchang 330006, China; 4206123070@email.ncu.edu.cn (S.S.); 409033240001@email.ncu.edu.cn (H.C.); 2 Jiangxi Research Institute of Ophthalmology and Visual Science, Nanchang 330006, China; 3 Jiangxi Clinical Research Center for Ophthalmic Disease, Nanchang 330006, China; 4 Jiangxi Provincial Key Laboratory for Ophthalmology, Nanchang 330006, China; 5 National Clinical Research Center for Ocular Diseases Jiangxi Province Division, Nanchang 330006, China

**Keywords:** NFS1, ferroptosis, iron homeostasis, ROS, lipid peroxidation

## Abstract

Ferroptosis is an iron-dependent form of regulated cell death (RCD) characterized by intracellular iron homeostasis disruption and lipid peroxide accumulation. It is involved in many pathological processes, including malignant tumors, cardiovascular diseases, inflammatory diseases, and mitochondrial disorders. Cysteine desulfurase (NFS1), a key enzyme in mitochondrial iron-sulfur (Fe-S) cluster biosynthesis, participates in regulating cellular ferroptosis by maintaining Fe-S cluster homeostasis and modulating the ACO1/IRP1 axis, the Xc^−^–glutathione (GSH)–glutathione peroxidase 4 (GPX4) axis, and the p53/*STAT* signaling pathway. When the function of NFS1 is abnormal, the intracellular free iron level is elevated, followed by reactive oxygen species (ROS) accumulation and lipid peroxidation. NFS1 expression exhibits significant variation across different tissues. Upregulation of NFS1 in tumors can enhance tumor cell resistance to ferroptosis; thus, it can promote tumor growth, drug resistance, and metastatic ability. Conversely, downregulation of NFS1 in cardiomyocytes and neurons exacerbates ferroptosis and causes functional impairment. Here, we systematically review recent advances in the molecular mechanisms of NFS1-mediated ferroptosis and its role in various disease models, intending to clarify key components in the upstream regulatory network of ferroptosis and explore the application value of NFS1 as a potential therapeutic target. The review shows that NFS1 plays an important role in cellular fate regulation, which has significant clinical application potential in the treatment of cancer and interventions for neurological and cardiovascular diseases. Therefore, it can provide a new theoretical basis and research direction for subsequent mechanism research and targeted therapeutic strategy development.

## 1. Introduction

Ferroptosis was firstly introduced by Dixon in 2012 [1] as a new type of regulated cell death (RCD) besides conventional types such as apoptosis and autophagy. The core characteristic of ferroptosis is the irreversible cellular damage caused by the accumulation of peroxidized lipids in the cell membrane [2,3,4]. The occurrence of ferroptosis is dependent on abnormalities in iron metabolism, disruption of the glutathione (GSH)—glutathione peroxidase 4 (GPX4) axis, and the accumulation of lipid peroxidation [5,6]. Free iron can produce hydroxyl radicals through the Fenton reaction, which damage polyunsaturated fatty acids (PUFAs) and induce lipid peroxidation. Concurrently, enzymes like ACSL4 and LPCAT3 facilitate the enrichment of PUFAs in membrane phospholipids and make them be susceptible to oxidative damage [7,8,9]. GPX4 is the key suppressor of ferroptosis by utilizing GSH to reduce lipid peroxides into lipoalcohols, thus maintaining cell membrane stability and integrity [10,11]. The activity of GPX4 depends on cellular uptake of cysteine (Cys), and the Xc^−^ system (SLC7A11/SLC3A2) is the main way to mediate the transport of Cys input. Nevertheless, P53 and other upstream regulators can reduce the input of Cys by impairing the expression of SLC7A11 [12,13,14]. This will weaken the antioxidant effect of GPX4 and then lead to reactive oxygen species (ROS) accumulation, finally enhancing the cellular susceptibility to ferroptosis [15,16]. In addition, cysteine desulfurase (NFS1) and its chaperone proteins ISCU, ISD11, and ABCB7 are involved in iron-sulfur (Fe-S) cluster synthesis and homeostasis, which in turn indirectly regulate the cellular ferroptosis thresholds and ROS buffering capacity [17,18]. In experimental studies, ferroptosis can be induced by small-molecule compounds. For instance, Erastin blocks cysteine uptake by inhibiting the Xc^−^ system [19,20], while RSL3 directly suppresses GPX4 activity [21,22]. Correspondingly, radical scavengers like Ferrostatin-1 and Liproxstatin-1 can effectively inhibit ferroptosis by trapping lipid peroxidation intermediates [23,24,25,26]. Morphologically, the most obvious ultrastructural changes in ferroptotic cells are found in mitochondria, including increased density of mitochondrial membrane, decreased or disappeared cristae, ruptured external membrane, and decreased volume of mitochondria [27,28,29]. In summary, ferroptosis is a type of RCD driven by the synergistic actions of iron-dependent ROS production and lipid peroxidation accumulation. Its distinctive molecular mechanisms offer new insights into the regulation of cellular fate and provide potential therapeutic targets for developing treatment strategies against tumors, cardiovascular diseases, and mitochondrial disorders.

In the multi-layered regulatory network of ferroptosis, the upstream regulators are increasingly attracting attention along with the classical GPX4 and System Xc^−^ [30,31]. Among these, the abnormal expression of NFS1 in diseases gradually shows its critical function in pathological ferroptosis. NFS1 is a highly conserved mitochondrial enzyme and mainly exists in the mitochondrial matrix. It is the rate-limiting enzyme in Fe-S cluster synthesis and performs irreplaceable functions by regulating ACO1/IRP1 status, maintaining homeostasis of the antioxidant system, and ensuring mitochondrial energy metabolism [32,33]. In the last few years, researchers have gradually localized NFS1 as an upstream node in ferroptosis regulatory networks and revealed its multidimensional effects in cell fate determination by integrating iron homeostasis, oxidative stress, and lipid metabolism [34,35]. When NFS1 is absent or dysfunctional, the Fe-S cluster synthesis is impaired, and then ACO1 will be converted to IRP1 subsequently. TfR1 will be upregulated and FTH1 will be downregulated consequently, which will raise the free iron level and activate the iron starvation response. Meanwhile, antioxidant enzymes like GPX4 are suppressed, resulting in excessive ROS accumulation. Finally, the cell will be triggered into ferroptosis [36]. Additionally, downregulation of NFS1 will activate the p53 pathway and inhibit the transcriptional activity of SLC7A11, impair the function of the Xc^−^ system, and restrict the cysteine uptake and GSH synthesis. The activity of GPX4 will be reduced finally, and lipid peroxidation accumulation will be enhanced. Ultimately, it will increase ferroptosis susceptibility. In diseases, the abnormal expression of NFS1 presents tissue-specific dual effects. In tumor cells, NFS1 is commonly overexpressed to maintain the Fe-S cluster synthesis and enhance the ferroptosis resistance. This can then promote drug resistance and metastasis, as observed in colorectal cancer (CRC) [34], lung cancer (LC) [36], gastric cancer (GC) [37], and hepatocellular carcinoma (HCC) [38]. However, in the cardiovascular system, NFS1 is often downregulated, and the deficiency of it can accelerate ferroptosis in cardiomyocytes, inducing myocardial injury and cardiomyopathy or cardiotoxicity [39,40,41]. In neurodegenerative diseases, the imbalance of NFS1 is also closely related to the disturbance of iron homeostasis and oxidative stress, suggesting potential involvement in the development of cognitive impairment and neuronal degeneration by regulating ferroptosis [32].

Based on the above discussion, it is evident that NFS1 acts as a key upstream regulator in the molecular mechanism of ferroptosis. Besides, NFS1 also acts as a crucial molecular node in the occurrence and progression of multiple diseases. Recent high-impact reviews by Ru et al. and Sun et al. in Signal Transduction and Targeted Therapy have comprehensively summarized the core regulatory networks of ferroptosis, including iron homeostasis, lipid peroxidation pathways, and the GPX4-centered antioxidant defense system [42,43]. However, these reviews primarily focus on the global framework of ferroptotic signaling and do not provide a systematic analysis of the Fe-S cluster biosynthetic machinery—particularly the cysteine desulfurase NFS1—and its role as an upstream metabolic checkpoint governing ferroptosis. To date, no review has examined ferroptosis from the perspective of NFS1 or integrated its functions in iron regulation, Fe-S cluster assembly, lipid ROS dynamics, and disease-specific ferroptosis susceptibility. Therefore, this review aims to fill this conceptual gap by providing the first comprehensive and mechanistic synthesis of NFS1-centered regulation of ferroptosis, with an emphasis on its roles in tumor biology, cardiovascular disorders, and mitochondrial diseases. Such an integrated perspective may offer new theoretical insights into basic research and inform the development of potential therapeutic interventions.

## 2. NFS1 and Iron Homeostasis Regulation

### 2.1. NFS1 Regulates the Fe-S–ACO1–IRP1 Axis

**Figure 1:**
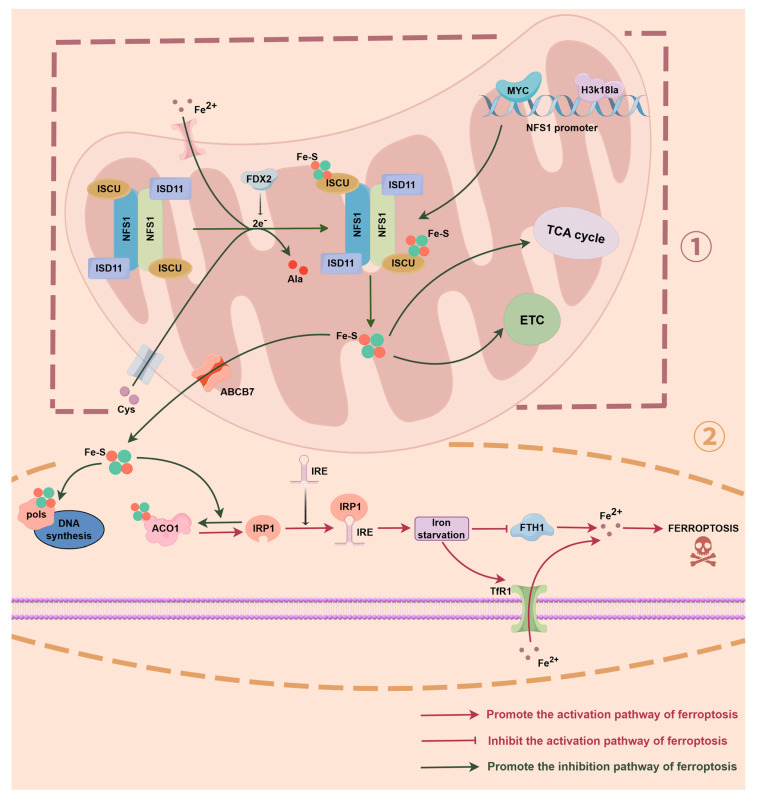
The molecular mechanism by which NFS1 drives Fe-S cluster biosynthesis and regulates the Fe-S-ACO1-IRP1 axis to maintain iron homeostasis (① Fe-S cluster synthesis and mitochondrial homeostasis maintenance: NFS1 resides in the mitochondrial matrix as the rate-limiting enzyme for iron-sulfur (Fe-S) cluster synthesis. It catalyzes Cys desulphurization in concert with ISCU, ISD11, and FDX2, supplying sulphur for Fe-S cluster assembly. The generated Fe-S clusters support critical metabolic processes, including DNA synthesis, electron transport, and the tricarboxylic acid cycle (TCA). NFS1 is upregulated under the influence of MYC and epigenetic modifications such as H3K18la. Its elevated expression enhances Fe-S supply, maintains ACO1 activity, and suppresses reactive oxygen species (ROS) accumulation, thereby counteracting ferroptosis. ② Imbalance in the Fe-S-ACO1-IRP1 axis and activation of ferroptosis induced by NFS1 deficiency: NFS1 deficiency disrupts Fe-S cluster assembly, converting ACO1 into IRP1. This mediates TfR1 upregulation and FTH1 downregulation, leading to excessive iron uptake and free Fe^2+^ accumulation. Excess Fe^2+^ generates ROS via the Fenton reaction, triggering lipid peroxidation and promoting ferroptosis.

**Figure 2:**
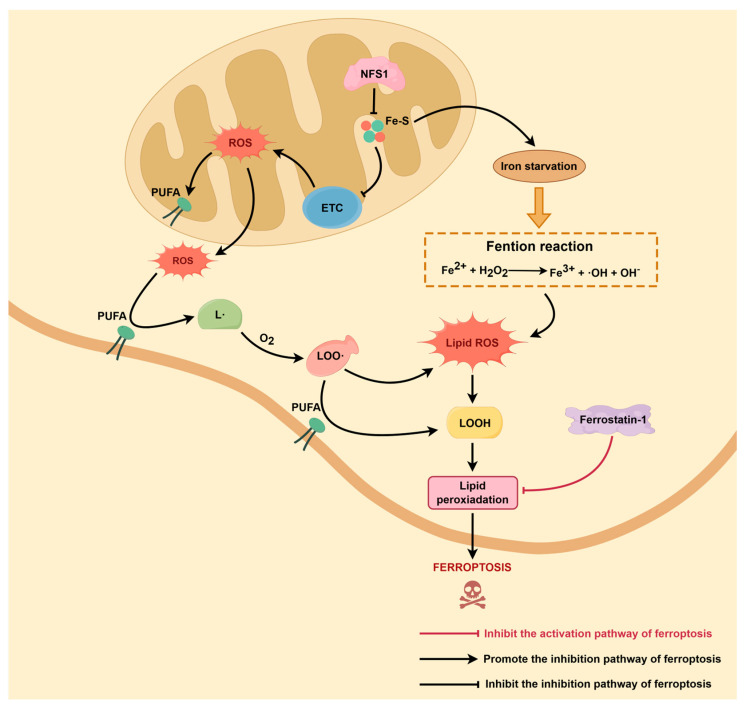
Molecular mechanism by which NFS1 deficiency causes ROS accumulation and promotes lipid peroxidation to induce ferroptosis. The molecular hallmark of ferroptosis is the excessive accumulation of membrane lipid peroxides, particularly phospholipid peroxidation products of PUFAs. When exposed to ROS, PUFAs can induce radical chain oxidation reactions of PUFAs, to produce lipid radicals (L•), lipid peroxy radicals (LOO•), and lipid peroxides (LOOH), disrupting the membrane structure and inducing cell death. NFS1 deficiency can disrupt the Fe-S cluster-dependent mitochondrial electron transport, causing abnormal ROS accumulation and enhancing lipid peroxidation reactions, ultimately accelerating ferroptosis.

Fe-S clusters serve as essential cofactors for at least 48 enzymes involved in fundamental cellular functions such as DNA synthesis and mitochondrial respiration, which encompasses the electron transport chain (ETC) and the tricarboxylic acid cycle (TCA). ACO1, as a prototypical Fe-S cluster-dependent enzyme, catalyzes citrate isomerization in its active form under normal conditions, participating in the TCA [44]. NFS1, located in the mitochondrial matrix, is the core rate-limiting enzyme catalyzing Fe-S cluster synthesis. By catalyzing the desulphurization of cysteine to provide a sulphur source, it participates in the assembly of Fe-S clusters, thereby maintaining the structural integrity and functional stability of mitochondrial and intracellular Fe-S proteins. This process is a key factor in ensuring the normal functioning of cellular metabolism and redox reactions [45,46,47]. Furthermore, NFS1 is directly regulated by the transcription factor MYC. In CRC models, MYC binds to its promoter to enhance NFS1 expression. Clinical CRC samples also demonstrate a significant positive correlation between NFS1 and MYC expression. High NFS1 expression is closely associated with chemotherapy resistance and poor prognosis [34] (as shown in Figure 1, ①). When the NFS1 function is impaired or its expression is downregulated, Fe-S cluster biosynthesis is disrupted. ACO1 loses its Fe-S domain and thus its enzymic activity, converting to IRP1 which binds to iron response elements (IREs) within mRNA, thereby activating the expression of iron metabolism-related genes. Upregulation of TfR1 increases iron uptake, whilst downregulation of FTH1 reduces iron storage, thereby disrupting cellular iron homeostasis [34,36]. At this juncture, cells ‘perceive’ an iron-deficient state, yet actual free iron levels ultimately rise, establishing the molecular basis for Fenton reactions and ferroptosis (as shown in Figure 1, ②). Moreover, to further provide a systematic overview of the major Fe-S proteins influenced by NFS1-dependent sulfur donation, we summarized these proteins and their functional characteristics (as shown in Table 1).

Lin et al. identified NFS1 as a key regulator of oxaliplatin sensitivity in CRC cells through *CRISPR-Cas9* metabolic enzyme genome screening. NFS1 deficiency markedly enhances oxaliplatin-induced cytotoxicity, primarily by altering cellular iron load through ACO1-IRP1 conversion, thereby increasing susceptibility to ROS and chemotherapy-induced damage. Conversely, NFS1 overexpression can preserve Fe-S cluster and ACO1 activity, and promote cell defense against oxidative stress [34]. In lung cancer models, NFS1 was found to undergo gene amplification and transcriptional activation in lung adenocarcinoma patients. The overall amplification rate of NFS1 was 38%, and its expression was particularly high in early-stage tumors and well-differentiated regions. This reflects that NFS1 might be a metabolic adaptation factor under screening pressure for pulmonary hyperoxic exposure [36]. When NFS1 was knocked down, Fe-S cluster synthesis was impaired, leading to cytoplasmic ACO1 inactivation and conversion to IRP1. Subsequently, the iron uptake was upregulated, while the iron storage was inhibited, and iron starvation responses as well as free iron accumulation were triggered. In hepatocellular carcinoma models, it was found that heat stress (HS) induced by microwave ablation (MWA) promoted glycolytic reprogramming in HCC cells. This was manifested by the upregulation of key enzymes (HK1, PFKL, etc.) and markedly increased intracellular lactate. Apart from being a metabolic byproduct, lactate could also drive epigenetic changes, most notably the significant enrichment of H3K18la. ChIP-seq confirmed that H3K18la accumulated in the promoter region of NFS1, and directly upregulated its transcription, which further enhanced Fe-S cluster synthesis, stabilized iron-dependent enzyme activity, and then maintained redox balance and inhibited ferroptosis (as shown in Figure 1, ①). Concurrently, this study also found that the intrinsic resistance of HCC to oxaliplatin partially depended on the NFS1-mediated suppression of ferroptosis. In both in vivo and in vitro experiments, the combined NFS1 inhibition with oxaliplatin significantly enhanced cytotoxicity and inhibited metastasis [38]. It is worth noting that the role of NFS1 is not limited to tumors, as it also plays a crucial role in hereditary diseases. In conditions such as Friedreich ataxia, NFS1 dysfunction impairs Fe-S cluster assembly. This further aggravates mitochondrial iron accumulation and oxidative damage, which provides a theoretical basis for the molecular mechanisms underlying these disorders [32]. In summary, NFS1 maintains the dynamic equilibrium of iron metabolism by regulating the Fe-S-ACO1-IRP1 signaling axis. When its function is disrupted, the precise regulation of iron metabolism is broken, inducing abnormal iron responses and the premature onset of ferroptosis.

Although NFS1 is predominantly localised in mitochondria, emerging evidence indicates the existence of a nuclear pool of NFS1. In yeast, Nfs1p contains a functional nuclear localization signal, and loss of its nuclear targeting results in loss of cell viability, suggesting that nuclear Nfs1p performs an essential function independent of mitochondrial Fe-S synthesis [48,49]. Similar observations have been made in protozoa, where NFS1 is found in the nucleus, and its depletion is lethal [50]. Functionally, non-mitochondrial NFS1 contributes sulphur for multiple extra-mitochondrial sulphur-dependent processes, such as MOCS3-mediated molybdenum cofactor biosynthesis and tRNA 2-thiolation [51,52]. While these functions do not constitute direct evidence for nuclear Fe-S cluster assembly, current models propose that nuclear NFS1 may facilitate localized sulfur provision that complements the cytosolic iron–sulfur assembly (CIA) machinery during the maturation of nuclear Fe-S proteins involved in DNA replication and repair [53]. Mitochondria-derived sulfur intermediates are thought to be transferred to cytosolic and nuclear Fe-S targets, while nuclear NFS1 may enhance local Fe-S delivery during DNA replication and repair, thereby linking Fe-S metabolism with genome stability.

**Table 1:** Major Fe-S cluster–containing proteins supported by NFS1-mediated sulfur donation.

Protein	Cellular Location	Function	NFS1 Role	References
ISCU	Mitochondria	Scaffold protein for Fe-S cluster assembly	Supply sulphur atoms for Fe-S cluster assembly	[18]
ABCB7	Mitochondria	Fe-S cluster export to cytosol	Provide Fe-S cluster precursors for ABCB7	[54,55]
ACO1/IRP1	Cytosol	Iron regulation via IRE binding	Provide Fe-S clusters, determining the functional state of ACO1	[34,36]
DNA polymerase	Nucleus	DNA replication fidelity	Generates Fe-S precursors for CIA-mediated assembly, indirectly supporting DNA polymerase	[56,57]
NDUFS1	Mitochondria	Complex I subunit in electron transport chain	NFS1 initiates Fe-S cluster formation required for ETC function	[58]
FDX1/FDX2	Mitochondria	Electron donors for Fe-S biosynthesis	NFS1-generated sulfur supports Fe-S cluster transfer	[59,60]
LIAS	Mitochondria	Lipoic acid synthesis	Supply sulphur to maintain the assembly of Fe-S clusters in LIAS	[61,62]

### 2.2. Free Iron Accumulation Drives the Fenton Reaction

In ferroptosis, the abnormal accumulation of free Fe^2+^ within cells is a prerequisite for the activation of the Fenton reaction. Since free Fe^2+^ could react with endogenous H_2_O_2_ to produce highly reactive hydroxyl radicals (•OH), in addition to activating the classic Fenton reaction cascade, it could also disturb the intracellular redox homeostasis by continuously producing ROS. Finally, it would induce the accumulation of lipid peroxidation products and cell membrane damage to induce ferroptosis [63] (as shown in Figure 2). Using the Mito-FerroGreen fluorescent probe, She et al. found that mitochondrial Fe^2+^ content was remarkably increased in NFS1 deficient models, implying that NFS1 dysfunction would directly expose mitochondria to iron-dependent oxidative stress [39]. In addition to the increase of local ROS generation, the mitochondrial-specific Fe^2+^ buildup could also cause a change of the mitochondrial membrane potential, which could promote the cross-activation of ferroptotic and apoptotic signaling pathways. While using the FerroOrange probe, Huang et al. further discovered that NFS1 knockout can also increase the content of free Fe^2+^ in both cytoplasm and mitochondria, generating dual oxidative stress. This provides the molecular basis for the increase of the Fenton reaction and propels lipid ROS accumulation beyond an irreversible level, promoting ferroptosis from its latent phase into execution [38]. Moreover, Fujihara et al. also indicated that NFS1 deficiency was accompanied with an abnormal increase in intracellular Fe^2+^ level and a substantial rise in ROS, which could directly activate ferroptosis [64]. In summary, NFS1 is responsible for the maintenance of a stable level of Fe-S cluster synthesis and functions as a barrier to suppress the accumulation of free iron and restrict the occurrence of Fenton reaction-driven oxidative chain reaction. Based on the above studies, we could conclude that NFS1 deficiency disrupts the assembly of the Fe-S cluster, leading to the intracellular accumulation of free Fe^2+^. Then, it activates the Fenton reaction to induce sustained ROS production, which would further induce an explosive accumulation of lipid peroxides and ultimately trigger ferroptosis. This process not only identifies the critical oxidative step in ferroptosis, but also reveals the key role of NFS1 in iron homeostasis and cellular fate.

## 3. NFS1 and ROS Plus Lipid Peroxidation

### 3.1. NFS1 Regulates Mitochondrial ROS Production

NFS1 is a core enzyme in the Fe-S cluster biosynthesis pathway, localized within the mitochondrial matrix. Its defects are found to be capable of triggering abnormal increases in mitochondrial-derived ROS. Fe-S clusters are widely distributed in many kinds of mitochondrial enzyme complexes, particularly complex I, II, and III of the ETC, and are crucial for maintaining mitochondrial electron transport stability and regulating ROS production [65]. Downregulation or impaired function of NFS1 can cause failure in Fe-S cluster synthesis and ETC function, and induce electron leakage. Finally, substantial ROS such as superoxide anion (O_2_^−^) are accumulated within mitochondria. Subsequently, oxidative stress and cellular damage are triggered by this high level of ROS [66,67]. Additionally, NFS1 deficiency has been further proven to have a specific influence on mitochondrial ROS. Jiang et al. also found that NFS1 knockdown could significantly increase the ROS and malondialdehyde levels in GC cells, which indicated an increase in oxidative stress [37]. Fujihara et al. employed MitoSOX staining to find that NFS1 deficiency could lead to respiratory chain complex dysfunction by decreasing Fe-S cluster stability. Finally, these reseachers found that NFS1 deficiency could increase the intracellular ROS level and induce apoptosis [64]. Wang et al. further confirmed via MitoSOX assay that the ROS accumulation was significantly enhanced in the NFS1 knockdown group, accompanied by mitochondrial DNA damage and cell death. All these results demonstrated that NFS1 is essential for maintaining mitochondrial redox homeostasis [40].

Furthermore, NFS1 deficiency also could impair the maintenance of mitochondrial membrane potential, and the mitochondrial membrane permeability was changed. Subsequently, ROS further releases and induces apoptosis or ferroptosis. JC-1 probe analysis was employed to find that NFS1 deficiency could significantly decrease mitochondrial membrane potential and ATP levels. It indicated that the mitochondrial energy homeostasis was affected [38]. This ‘metabolic crisis’ state would accelerate ROS accumulation, induce mitochondrial swelling and cristae disruption, which present typical phenotypes of ferroptosis. In this process, ROS not only behaves as the oxidative stressor, but also acts as the signaling molecule leading to lipid peroxidation, thus serving as a crucial upstream driver of ferroptosis. It is noteworthy that O_2_^−^ accumulated within mitochondria can rapidly react with nitric oxide (NO) to form the potent oxidant peroxynitrite (ONOO^−^). ONOO^−^ not only directly mediates oxidative damage to mitochondrial membrane lipids but also nitrates critical tyrosine residues in complexes I and III, thereby compromising the stability of the ETC. This disruption leads to sustained ROS production, establishing a self-amplifying vicious cycle of ONOO^−^ accumulation [68,69]. In animal models, inhibition of ONOO^−^ has further validated its pathological role. For instance, the mitochondria-targeted antioxidant MitoQ reduces protein nitration, preserves complex activity, and restores mitochondrial membrane potential and ATP production, demonstrating that preventing ONOO^−^ accumulation can effectively mitigate age- or pathology-related mitochondrial dysfunction [70]. Abnormal ONOO^−^ accumulation is considered a central mediator of mitochondrial structural damage, increased membrane permeability, and accelerated lipid peroxidation following NFS1 dysfunction. In summary, NFS1 exerts its function in suppressing mitochondrial ROS production by maintaining the function of the Fe-S cluster-dependent ETC and antioxidant system. In addition to being early molecular processes brought on by ferroptosis, impairments in its expression or function may also be a key molecular basis for the impairment of mitochondrial homeostasis in highly metabolically active cells, such as tumor cells and cardiomyocytes.

### 3.2. Lipid ROS Accumulation Is Key to the Ferroptosis Initiation

The most prominent feature of ferroptosis is the abnormal accumulation of membrane lipid peroxides, and the generation of phospholipid peroxides has been proven to be one of the most important events driving ferroptosis. When ROS initiate the radical chain reactions from the PUFA phospholipid moiety, ROS attack PUFAs to form lipid radicals (L•), which subsequently combine with oxygen to generate lipid peroxy radicals (LOO•) and lipid peroxides (LOOH). These compounds then disrupt the membrane structure and induce irreversible cell death [71]. When NFS1 is absent, this chain reaction becomes uncontrolled and intensifies, resulting in a significant elevation of lipid ROS levels [72] (as shown in Figure 2). Huang et al. used BODIPY 581/591 C11 dye to detect lipid ROS levels. They found that there was substantial intracellular lipid ROS accumulation following NFS1 knockdown, which was highly correlated with ferroptosis phenotypes. This experiment proved that NFS1 played an important role as a ‘braking factor’ in ferroptosis [38]. It is particularly noteworthy that ONOO^−^ functions as a crucial ‘amplifier’ within the lipid peroxidation chain reaction, markedly accelerating the accumulation of lipid-derived ROS under conditions of NFS1 deficiency. ONOO^−^ can directly abstract bis-allylic hydrogen atoms from PUFA, thereby initiating lipid peroxidation in an iron-independent manner. Its decomposition generates highly reactive radicals, including •OH and nitrogen dioxide radical (•NO_2_), which rapidly oxidize PUFAs to produce large amounts of LOO• and LOOH, further exacerbating membrane lipid damage [73]. In addition, accumulating evidence indicates that ONOO^−^ can activate lipoxygenases such as 15-LOX through nitration, shifting lipid peroxidation from nonspecific free-radical oxidation toward targeted enzymatic oxidation and thereby promoting ferroptotic progression [74,75]. Thus, ONOO^−^ is considered a central chemical driver of uncontrolled lipid ROS accumulation resulting from NFS1 loss, ultimately shaping cellular sensitivity to ferroptosis.

Moreover, iron load generates highly reactive ROS such as •OH via the Fenton reaction, and then directly accelerates lipid peroxidation. With C11-BODIPY probes, She et al. observed significantly elevated lipid ROS level under iron-loaded conditions alongside heightened ferroptosis pathway activity [39]. LC models further demonstrated that although NFS1 deficiency alone was insufficient to directly trigger ferroptosis, it markedly heightens the sensitivity of LC cells to exogenous ROS stress or GSH depletion, accelerating lipid peroxide accumulation and inducing ferroptosis. This effect was rescued by Ferrostatin-1, proving that it was ferroptosis dependent [36]. Besides, in the HCC model, NFS1 overexpression enabled HCC cells to maintain low ROS, Fe^2+^, and lipid peroxidation levels under hyperoxia and stress conditions, and markedly promoted migration, invasion, and epithelial-mesenchymal transition. While NFS1 knockout could restore the ferroptosis phenotype, reduced N-cadherin and vimentin expression, and markedly decreased the number of lung metastatic foci. All these results suggested that NFS1 played a crucial regulatory role in tumor invasion and metastasis by modulating lipid ROS levels [38]. In summary, lipid ROS not only are direct executors of ferroptosis but also are terminal effector markers of signaling imbalance mediated by NFS1 dysfunction, and have potential value in prediction and intervention.

## 4. Interaction Between NFS1 and Key Ferroptosis Pathways

### 4.1. NFS1 Regulates the GPX4-GSH-Xc^−^ System Axis

**Figure 3:**
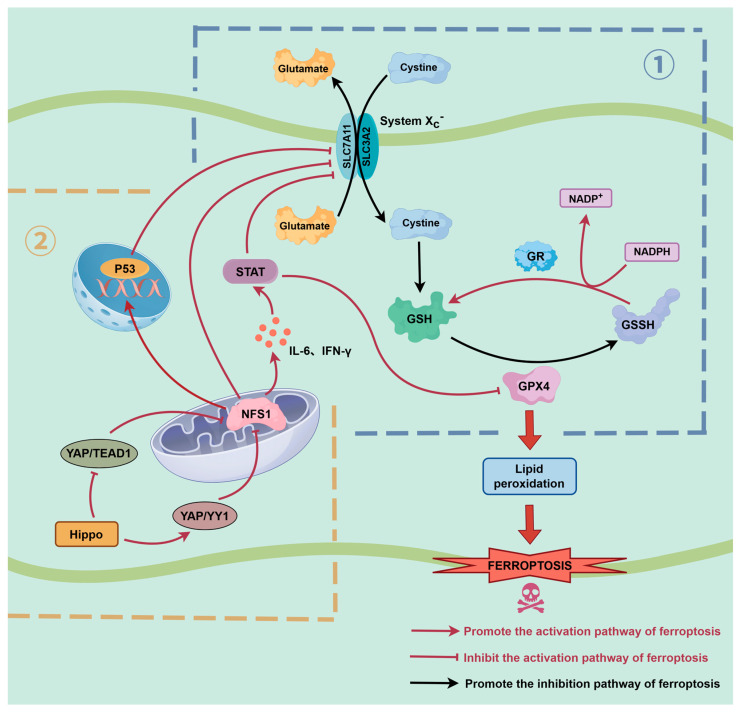
NFS1-Hippo, p53, and *STAT* signaling axes regulate the ferroptosis mechanism mediated by the System Xc^−^- glutathione (GSH) -glutathione peroxidase 4 (GPX4) pathway. (① System Xc^−^-GSH-GPX4 antioxidant defense: System Xc^−^ comprises SLC7A11 and SLC3A2, which maintain intracellular GSH synthesis by promoting extracellular Cys uptake and Glu efflux. As a key substrate for GPX4, GSH participates in lipid peroxide clearance, thereby inhibiting ferroptosis. ② Upstream regulation and signaling network of NFS1: Activation of the Hippo pathway promotes *YAP-YY1* binding, weakening *YAP/TEAD1* complex formation and downregulating NFS1 expression. NFS1 deficiency induces mitochondrial oxidative stress and Fe-S cluster imbalance, activates *STAT* signaling and inflammatory factor secretion like IL-6 and IFN-γ, and upregulates p53. Activated p53 then inhibits SLC7A11 transcription, thereby impairing Xc^−^-mediated Cys uptake, which reduces GSH levels and GPX4 activity, and promotes lipid peroxidation.

GPX4 is an indispensable enzyme in protecting cells from lipid peroxidation. Its activity depends on GSH, while GSH synthesis relies on the sufficient content of Cys [76,77,78]. The Xc^−^ system is composed of SLC7A11 and SLC3A2, which is a Cys/glutamate (Glu) exchanger located in the cell membrane. It facilitates the inward transport of extracellular Cys, serving as a key upstream part in maintaining the synthesis of GSH and the stability of GPX4 activity [79,80] (as shown in Figure 3, ①).

Previous studies have demonstrated that NFS1 deficiency or downregulation could disrupt iron homeostasis and increase ROS levels. Subsequently, transcription and expression of SLC7A11 were decreased, and cysteine uptake was inhibited. Further analysis revealed that GPX4 expression was dampened and its protein was stabilized poorly. Breaking of the antioxidant barrier ultimately leads to substantial accumulation of lipid peroxides, initiating ferroptosis [39,81]. More importantly, ONOO^−^ can nitrate or oxidize the selenocysteine residue of GPX4, directly suppressing its enzymatic activity and thereby amplifying the lipid ROS accumulation driven by NFS1 dysfunction [82,83]. This positions ONOO^−^ as a critical chemical mediator linking impaired NFS1 function to GPX4 inactivation. In addition, ONOO^−^ can oxidize GSH and inhibit system Xc^−^ activity, leading to reduced cystine uptake. Such interference renders the NFS1–GPX4–GSH–system Xc^−^ axis highly vulnerable to disruption, further accelerating ferroptotic progression. In CRC models, NFS1 has been found to co-express with other antioxidant enzymes (such as the GPX family), suggesting its critical role in maintaining redox homeostasis and promoting tumor drug resistance [34]. Similarly, in LC models, konckdown of NFS1 dramatically increased cellular sensitivity to GPX4 inhibitor RSL3, and simultaneously enhanced vulnerability to the Xc^−^ system inhibitor Erastin and the cysteine depletant cyst(e)inase. This also demonstrates that NFS1 constructs a multi-layered anti-ferroptotic protective network through the GPX4-GSH-Xc^−^ system axis [36]. In non-neoplastic diseases, NFS1 deficiency also exhibits significant consequences. In neonatal mice model of septane induced neurotoxicity, deficiency of NFS1 resulted in impaired the function of multiple Fe-S dependent enzymes (e.g., ACO2, complexes I–III). Impairment of tricarboxylic acid cycle was observed, and efficiency of ETC was dampened, leading to enhanced mitochondrial ROS production. The accompanied excessive accumulation of Fe^2+^ not only drove the Fenton reaction but also disrupted the balance of the GSH-GPX4 antioxidant system, exacerbating lipid peroxidation damage. Interestingly, in Parkinson’s disease models, iron load correlates strongly with neuronal death in the substantia nigra, suggesting downregulation of NFS1 may represent a molecular link between ferroptosis and neurodegeneration [32].

Considering the central role of NFS1 in maintaining antioxidant defense and iron homeostasis, its upstream regulation is of great importance. In recent years, the Hippo signaling pathway has been gradually proven to play a key role in regulating the expression of NFS1. As a core effector of the Hippo signaling pathway, Yes-associated protein (YAP) not only participates in cell proliferation, stress responses, and organ size regulation, but is also closely associated with ferroptosis. For the first time, a cardiovascular model has demonstrated that Hippo pathway activation significantly downregulates NFS1 expression by enhancing *YAP* binding to *YY1* while weakening *YAP* binding to *TEAD1* [39] (as shown in Figure 3, ②). In a mouse model of cardiomyopathy induced by anti-myosin antibodies, significantly elevated *MST1* and *LATS1* expression was observed in myocardial tissue, accompanied by enhanced *YAP* phosphorylation (inactivated state), indicating sustained Hippo pathway activation and suppression of *YAP* transcriptional function. Further molecular experiments demonstrated that *MST1* overexpression or *YAP* knockout both led to decreased NFS1 mRNA and protein levels, while *MST1* inhibitors reversed this effect. ChIP and dual luciferase assays further prove that the YAP-TEAD complex directly binds to the NFS1 promoter region and promotes its transcription, confirming NFS1 as a direct downstream target gene of *YAP*. These results demonstrated that the Hippo pathway could directly regulate NFS1 expression by inhibiting *YAP* activity, which constituted an upstream control layer for the systemic Xc^−^-GSH-GPX4 antioxidant axis and further influencing cellular sensitivity to ferroptosis. In the tumor microenvironment, changes in cell density and contact inhibition can regulate ferroptosis tolerance by modulating the Hippo-YAP pathway [84]. Meanwhile, the Hippo-YAP pathway further shapes susceptibility to ferroptosis by altering cell membrane composition through regulating fatty acid synthesis and cholesterol metabolism. Obviously, the Hippo-YAP-NFS1-ferroptosis axis not only possesses significant implications in cardiovascular diseases but may also play a vital role in tumor adaptive survival and metabolic reprogramming. Therefore, NFS1 could indirectly protect the activity of System Xc^−^ and GPX4-dependent defense against lipid peroxidation by maintaining Fe-S cluster synthesis, stabilizing mitochondrial function, and preserving iron metabolic equilibrium, which establishes a multi-layered regulatory network for ferroptosis. Dysfunction of NFS1 might represent the initial molecular event in the imbalance of the System Xc^−^-GPX4 axis, offering a novel molecular basis for future targeted interventions in ferroptosis.

### 4.2. Cooperative Regulation of the NFS1 and p53 Pathways

NFS1 and p53 exhibit closely synergistic effects in regulating ferroptosis sensitivity. As a classic tumor suppressor protein, p53 exerts a central regulating role in oxidative stress, mitochondrial function maintenance, and multiple cell death pathways including ferroptosis [15,85,86]. Its typical mechanism involves inhibiting the transcriptional activity of SLC7A11, which weakens Xc^−^-mediated Cys uptake and further inhibits GSH synthesis. Downstream, it reduces the activity of GPX4, ultimately promoting lipid peroxidation accumulation and triggering ferroptosis [87,88].

Previous studies have shown that downregulation of NFS1 expression leads to activation of the p53 pathway, which in turn inhibits SLC7A11 expression. This impairs the transport function of System Xc^−^ for cysteine, and then further restricts the supply of intracellular antioxidant substrates. Consequently, the content of GSH decreases, and the reductive activity of GPX4 is lost. Finally, the accumulation of lipid peroxides will be accelerated and ferroptosis will be induced (as shown in Figure 3, ①). Further KEGG pathway enrichment analysis revealed that genes highly correlated with NFS1 expression were significantly enriched in the p53 signaling pathway. This suggests NFS1 may indirectly influence ferroptosis susceptibility by regulating p53 activity, thereby affecting SLC7A11 transcription levels and Xc^−^ system function [89]. This synergistic method shows that NFS1 may act as an upstream node in oxidative stress signaling, and then help to tune the p53 signaling pathway and so alter ferroptosis susceptibility, along with regulating iron metabolism by preserving Fe-S cluster balance. Moreover, it is well known that p53 mutations are frequently observed in various tumors including GC, and the NFS1-p53 axis may constitute an important molecular basis for ferroptosis escape and chemotherapy resistance. This mechanism offers novel insights for targeting ferroptosis in antitumor therapies, suggesting that the NFS1-p53 axis may be considered as a potential therapeutic target. However, its specific molecular networks and regulatory details will need to be further explored through systematic experimental studies.

### 4.3. NFS1-STST Axis

NFS1 maintains the homeostasis of mitochondrial Fe-S cluster, and it also indirectly influences *JAK/STAT* signaling activation by modulating the intracellular ROS production and ferroptosis susceptibility. NFS1 deficiency induces dysfunction of mitochondrial ETC and increases ROS release. In addition to promoting lipid peroxide accumulation, this oxidative stress also acts as a signaling molecule to induce cytokine secretion (e.g., IL-6, IFN-γ), and further activate the *STAT* pathway (as shown in Figure 3, ①). In a GC model, Jiang et al. observed that the positive expression rate of NFS1 was remarkably higher in patient tissues (73.1%) than that in normal gastric mucosa (30.1%). This expression was closely associated with lymph node metastasis and advanced TNM stages. The patients with high expression of NFS1 presented markedly reduced overall survival (Cox regression HR = 1.612, *p* = 0.017), indicating NFS1 as an independent adverse prognostic factor. Functional assays showed that NFS1 knockdown could suppress proliferation, migration, and invasion of GC cells, as well as induce a typical ferroptosis phenotype such as a decrease of mitochondrial cristae, membrane densification, and an increase of ROS, Fe^2+^, and malondialdehyde levels, accompanied by downregulation of GPX4 and SLC7A11. Specifically, the ferroptosis inhibitor Ferristatin-1 partially rescued this phenomenon, while the apoptosis inhibitor Z-VAD-FMK and the necrosis inhibitor Nec-1 showed no significant effect, which indicated that NFS1 knockdown could mainly induce cell death in ferroptosis pathway. In MGC803 and SGC7901 cell lines, as well as corresponding nude mouse xenograft models, NFS1 deficiency could inactivate *STAT* signaling and suppress the malignant phenotype in GC cells. Mechanistically, studies revealed that NFS1 activates *STAT3* signaling, thereby upregulating GPX4 and SLC7A11 to reduce lipid peroxide accumulation, and maintaining cellular antioxidant capacity and iron homeostasis. The *STAT3* agonist colivelin could effectively alleviate the ferroptosis phenotype induced by NFS1 knockdown. Therefore, the NFS1-*STAT3* axis is an essential molecular switch for GC cells to resist ferroptosis [37]. These findings demonstrate that NFS1 functions not merely as a metabolic maintenance factor but profoundly influences cellular fate determination by regulating cytokine release and *STAT* signaling.

## 5. Research on NFS1 in Disease

### 5.1. Tumor

NFS1 exhibits persistent high expression in multiple solid tumors, including CRC, LC, GC, and HCC, and is recognized as a crucial molecular barrier enabling tumor cells to evade ferroptosis. As the rate-limiting enzyme in Fe-S cluster biosynthesis, NFS1 catalyzes Cys desulphurization, providing a critical sulphur source for Fe-S cluster assembly, which maintains the conformational stability and catalytic activity of numerous Fe-S dependent enzymes. This process efficiently inhibits lipid peroxidation events and aberrant ROS formation in addition to protecting mitochondrial oxidative phosphorylation and lipid metabolism balance. Therefore, it significantly enhances cellular antioxidant defense capability and further reduces the possibility of ferroptosis occurrence [90].

In CRC, NFS1 exhibits markedly elevated expression compared to adjacent normal tissue, and upregulates in patients with lymph node metastasis or recurrence, which suggests its important role in tumor progression and prognosis. According to current findings, elevated NFS1 expression is a major contributor to chemotherapy resistance and malignant development in addition to providing a molecular barrier that allows cancer cells to avoid ferroptosis [34]. Mechanistically, NFS1 suppresses the cytotoxic effects of oxaliplatin by maintaining Fe-S cluster assembly and redox homeostasis, as well as inhibiting ROS accumulation and PANoptosis. This process is further regulated by MYC-mediated transcriptional activation. In summary, NFS1 maintains CRC chemotherapy resistance and malignant progression by maintaining iron homeostasis and antioxidant defenses via the Fe-S-ACO1-GPX4 axis. Meanwhile, it simultaneously suppresses PANoptosis through phosphorylation-dependent regulation of ROS signaling. Therefore, it may serve as a potential biomarker to predict CRC chemotherapy sensitivity and as a target for therapeutic intervention.

In LC, especially in lung adenocarcinoma, the exact role of NFS1 is still very important, but its mode of action seems to be more related to the hyperoxic environment existing in lung tissue. NFS1 is constantly upregulated in lung tissue with increased partial pressure of oxygen, and represents a key barrier that enables tumor cells to survive in hyperoxic enviroment and escape ferroptosis. Under 21% oxygen conditions, NFS1 deficiency greatly inhibits the proliferation of LC cells, while its impact is relatively limited in 3% hypoxic environment, suggesting that NFS1 takes a central role in oxygen-dependent survival [36]. Trough maintaining the integrity of Fe-S dependent enzyme systems and iron homeostasis, while potently interacting with antioxidant systems, NFS1 establishes a defense network that enables tumor cells to resist oxidative stress and ferroptosis. The constant upregulation of NFS1 expression in lung adenocarcinoma reflects not only the selective pressure caused by hyperoxic exposure, but also its important role in tumor metabolic adaptation, tolerance, and therapeutic resistance, which makes NFS1 potentially can be used as a prognostic biomarker and target for individualized therapy.

NFS1 is similarly highly expressed in the GC and plays a central role in ferroptosis regulation, tumor malignant progression, and immune microenvironment remodeling. Jiang et al., based on TCGA and clinical samples, found that NFS1 overexpression was positively correlated with invasion depth, lymph node metastasis, and TNM staging, and constitutes an independent adverse prognostic factor. Functional enrichment analysis showed that NFS1 was involved in p53 signaling, platinum resistance, and ROS regulation. Through Immunological studies, it was found that high expression of NFS1 was correlated with reduced infiltration of effector immune cells and increased proportions of immunosuppressive cells, and was also exhibiting positive correlations with PD-L1 and CTLA-4 expression, which suggested that it promoted immune evasion. Clinical predictions further indicated that patients with low NFS1 expression exhibited greater sensitivity to PD-1/CTLA-4 inhibitors, whereas high expression might indicate resistance to immunotherapy [89]. In summary, NFS1 drives GC progression by inhibiting ferroptosis and remodeling the immune microenvironment concurrently, offering a potential target for combined ferroptosis induction and immunotherapy.

HCC ranks as the third leading cause of cancer-related mortality worldwide. Whilst MWA is an indispensable minimally invasive therapeutic approach for early-stage HCC, incomplete microwave ablation (IMWA) frequently induces sublethal HS, which results in the residual tumor cell migration and drug resistance. Huang et al. reported that HS enhanced lactate accumulation and further increased histone H3K18 lacylation [38]. Then, the increased histone H3K18 lacylation enhanced NFS1 transcriptional activity, boosting Fe-S cluster biosynthesis to maintain mitochondrial homeostasis and suppress ROS production. Consequently, HCC cells exhibited reduced lipid peroxidation, and ultimately, the tumor cell migration and chemotherapy resistance were enhanced. This study initially links lactate, a Warburg effect product, to histone lactylation and ferroptosis tolerance. It proposes a mechanism whereby H3K18la modulates NFS1 to resist ferroptosis and promote metastasis. Thus, it provides a molecular basis for HCC recurrence after IMWA. H3K18la and NFS1 emerge as potential prognostic markers and therapeutic targets. In the future, NFS1-targeting small-molecule inhibitors may be applied in preventing metastasis either alone or in combination with chemotherapy or ferroptosis inducers.

### 5.2. Cardiomyopathy

NFS1 is also important in mitochondrial homeostasis and antioxidant regulation in cardiomyocytes. As a mitochondrial cystathionine desulfurase, NFS1 provides sulfur for Fe-S cluster biosynthesis in cardiomyocytes and further maintains the activities of the ETC and multiple Fe-S dependent enzymes to ensure oxidative phosphorylation energy production and lipid metabolism homeostasis. Impaired NFS1 function disrupts Fe-S cluster assembly, and the activity of the mitochondrial respiratory chain is impaired. Then, cardiomyocytes present decreased ATP synthesis and enhanced ROS accumulation. This leads to oxidative stress and contractile dysfunction, which finally promotes the onset and progression of polymorphic cardiomyopathy.

Recently, studies have elucidated the specific mechanisms of NFS1 involved in cardiomyopathy. She et al. first proposed the Hippo-YAP-NFS1-ferroptosis axis, which linked NFS1 downregulation to dilated cardiomyopathy [39]. Then, Wang et al. demonstrated that NFS1 deficiency in diabetic cardiomyopathy impairs Fe-S cluster synthesis and triggers PARthanatos [40]. Overall, NFS1 functions as both an essential barrier against ferroptosis and a vital node preventing activation of non-canonical cell death pathways in cardiomyocytes. By preventing cardiomyocyte mortality via modulation of the Hippo-YAP axis, directly restoring NFS1 expression, or stabilizing Fe-S clusters, these discoveries not only enrich the molecular pathophysiology of cardiovascular diseases, but also provide novel strategies for myocardial protection. However, given that NFS1 can promote tumor tolerance, its systemic activation should be careful evaluated to balance cardiovascular benefits against oncogenic risks.

### 5.3. Mitochondrial Disorders

Loss-of-function mutations in NFS1 are the direct cause of two severe congenital mitochondrial disorders: compound oxidative phosphorylation deficiency type 52 (COXPD52) and type 19 (COXPD19). These disorders provide the strongest human genetic evidence that NFS1 is indispensable for Fe-S cluster biogenesis and mitochondrial respiratory function. Pathogenic variants, including missense and splice-site mutations, markedly reduce NFS1 enzymatic activity, leading to defective Fe-S cluster formation and impairment of multiple Fe-S-dependent mitochondrial enzymes, particularly complexes I–IV of the oxidative phosphorylation (OXPHOS) system.

COXPD52 is predominantly caused by biallelic pathogenic mutations in NFS1, among which the missense mutation c.215G>A (p.Arg72Gln) is the most frequent [91,92]. This mutation significantly decreases NFS1 protein stability and catalytic activity, resulting in impaired mitochondrial Fe-S cluster assembly and compromised OXPHOS function. Affected infants typically present in the neonatal period with hypotonia, lactic acidosis, respiratory failure, and multi-organ energy metabolism dysfunction, accompanied by a pronounced decrease in mitochondrial complex activity, especially in complexes II and III [93]. COXPD19, in contrast, is caused by homozygous mutations in the LYRM4 gene, which encodes ISD11, a critical NFS1-binding partner [94]. ISD11 forms a stable complex with NFS1, maintaining its proper conformation and participating in the early stages of Fe-S cluster assembly. The most commonly reported ISD11 homozygous mutation, R68L, destabilizes the NFS1–ISD11 complex, thereby inhibiting Fe-S cluster synthesis, promoting mitochondrial iron accumulation, and substantially reducing OXPHOS complexes I–IV activity [95]. Clinically, affected patients present with neonatal respiratory distress, hepatomegaly, severe lactic acidosis, and marked mitochondrial structural abnormalities. Collectively, COXPD52 and COXPD19 underscore the critical role of the NFS1–ISD11 complex in sustaining Fe-S cluster biogenesis and mitochondrial function.

Mechanistic studies suggest that impairment of the NFS1–ISD11 axis may sensitize cells to ferroptosis [96]. However, clinical and pathological evidence to date has not demonstrated overt ferroptotic phenotypes in COXPD52 or COXPD19 patients. Reported cases consistently describe severe mitochondrial structural defects, energy metabolism collapse, and lipid accumulation, yet alterations in canonical ferroptosis markers—such as GPX4 downregulation, ACSL4 upregulation, lipid peroxidation, or characteristic ferroptotic morphology—have not been observed. This discrepancy may be attributed to the rapid energy failure and cell death resulting from severe NFS1 deficiency, which could mask or preclude ferroptosis. Furthermore, ferroptosis may require partial rather than complete reduction of NFS1 activity for induction, a notion supported by evidence from select cellular and animal models [37].

In summary, COXPD52 and COXPD19, as human genetic models of NFS1–ISD11 complex deficiency, provide valuable insights into the interplay among Fe-S cluster biogenesis, mitochondrial oxidative metabolism, and cellular redox homeostasis. While existing data indicate that NFS1 dysfunction may create a permissive environment for ferroptosis, direct evidence for its involvement in the pathophysiology of these disorders remains lacking. Future studies should focus on assessing ferroptosis-related markers in patient-derived cells, induced pluripotent stem cell models, or postmortem tissues, including the expression of GPX4, SLC7A11, and ACSL4, cellular labile iron pool levels, and lipid peroxidation products. Such investigations will clarify whether ferroptosis contributes to NFS1-related mitochondrial diseases and further elucidate the intersection between Fe-S cluster biology and ferroptosis in human pathophysiology.

## 6. Discussion

Ferroptosis, as a distinct form of RCD, has been widely implicated in various pathological processes, including tumors, cardiovascular injury, andmitochondrial disorders. Its process is closely associated with the dynamic imbalance of iron homeostasis, oxidative stress, and lipid peroxidation [97,98]. As research advances, it has been gradually recognized that targeting downstream effector molecules alone may bring limited benefits in the long term. Consequently, exploring upstream regulatory nodes of ferroptosis becomes a novel research direction. Due to the unique molecular localization, NFS1 is presumably a potential target for ferroptosis regulation. Current intervention strategies for ferroptosis are mainly focused on downstream effector molecules, such as directly inhibiting GPX4 activity or blocking SLC7A11-mediated Cys uptake [99,100,101]. However, these approaches are often limited by the activation of compensatory pathways and the development of acquired tolerance [102,103]. In contrast, NFS1, as a cystathionine desulfurase, occupies the initial step of the Fe-S cluster biosynthetic pathway and serves as an ‘upstream convergence point’ for multiple ferroptosis defense axes [104,105]. Its catalytic activity not only determines the conformational stability of Fe-S dependent enzymes such as those in the ETC and tricarboxylic acid cycle, but also regulates the free iron pool and cellular iron starvation response via the ACO1/IRP1 axis. At the metabolic level, it indirectly supports the ‘energy-supplying-antioxidant’ capacity of anti-lipid peroxidation branches, including the GPX4-GSH-system Xc^−^ and mitochondrial inner membrane Dihydroorotate dehydrogenase (DHODH). From this perspective, targeting NFS1 holds promise for achieving systematic reprogramming of the three-dimensional network linking iron metabolism, oxidative phosphorylation, and lipid peroxidation. Within the tumor microenvironment [106,107], short-range or localized inhibition of NFS1 can form synthetic lethality effects with erastin, RSL3, cyst(e)inase, and radiotherapy or ROS inducers. This significantly enhances ferroptosis sensitivity and overcomes resistance barriers. Contrarily, in highly metabolically active tissues like myocardium [39], which are critically dependent on mitochondrial function, activating or stabilizing NFS1 may inhibit ferroptosis and mitochondrial dysfunction by enhancing Fe-S cluster homeostasis and mitochondrial integrity. This ‘protective’ regulation, based on tissue differentiation and disease progression, provides a clear biological lever for precision therapy.

As a rate-limiting step of cellular basal metabolism, the pharmacological regulation of NFS1 exhibits pronounced double-edged effects. On the one hand, systemic inhibition of NFS1 may induce on-target/off-tissue adverse reactions such as myocardial mitochondrial toxicity and metabolic collapse [40]. On the other hand, sustained activation may confer cellular survival tolerance in certain tumor contexts, which promotes immune evasion and drug resistance development [36,108]. More interestingly, NFS1 inhibition rapidly triggers the IRP1-dependent iron starvation response and compensatory iron uptake [109], which paradoxically expands the free iron pool in the short term and amplifies Fenton reaction-driven lipid peroxidation. Although it helps tumor cell clearance, it greatly increases the risk of oxidative damage in normal tissues. Within the myocardial system, the Fe-S network is highly dependent on NFS1. If there is no compensation, it may transition into non-canonical cell death pathways such as PARthanatos, leading to mechanism drift and unpredictable toxic consequences. Consequently, tissue selectivity, the time-dose window, and reversibility will constitute the three primary safety thresholds for NFS1 pharmacological intervention. In terms of drug modalities, tumor therapy may explore reversible small-molecule inhibitors, PROTAC/molecular glue-mediated tumor-selective degradation [65], and programmed combination strategies with cisplatin, radiotherapy, or ferroptosis inducers [110,111,112]. For cardiovascular protection, NFS1 stabilizers or protein-protein interaction enhancers may be considered, or mRNA therapies and AAV9 myocardial-targeted expression could achieve short-term “cardio-preservation” effects [113,114,115]. Regarding delivery strategies, tumor-targeting nanodelivery systems or liver-targeting GalNAc platforms combined with local administration may be employed [38,116,117,118,119], alongside sequential pulsed dosing to extend therapeutic windows [120]. For complementary companion diagnostics and pharmacodynamic assessment, a ‘three-tiered indicator system’ should be established. Firstly, at the target level, evaluation can be achieved by monitoring NFS1 protein expression and activity, alongside the activity profiles of Fe-S dependent enzymes (e.g., ACO2, SDHB, NDUFS1) [121]. Second, the pathway layer can be dynamically tracked through indicators, including free iron pool levels, GSH/GSSG ratio, lipid ROS, mitochondrial membrane potential, and oxygen consumption flux. Third, the phenotypic layer relies on reversible rescue assays using Fer-1/Lip-1, alongside histological ferroptosis markers and myocardial function readouts for determination [122]. In clinical trial design, an approach combining enrichment enrolment with adaptive randomization may be employed [123,124]. In terms of oncology research, we should focus on screening ‘NFS1-high expression, Fe-S-dependent, or antioxidant-dependent’ subpopulations, and exploring combination with low-dose platinum or systemic Xc^−^ inhibitors. Within the cardiomyopathy field, we should adopt short-course myocardial-directed interventions during the perioperative or acute injury phase, and strictly monitor the oncological risk. This will achieve dual gatekeeping for organ preservation and tumor safety.

Another form of regulated cell death, tightly linked to Fe-S cluster homeostasis—cuproptosis—also warrants attention. Cuproptosis is initiated when mitochondrial labile copper directly binds to lipoylated proteins, inducing the aggregation and functional collapse of Fe-S–dependent mitochondrial enzymes [125,126]. Consequently, impaired NFS1 activity or insufficient sulfur supply accelerates the loss of Fe-S–containing proteins, thereby substantially increasing cellular susceptibility to cuproptosis. In addition, cuproptosis exhibits regulatory overlap with ferroptosis, as both processes are shaped by mitochondrial metabolic status, metal ion homeostasis, and redox stress [127]. Incorporating cuproptosis into the downstream landscape of NFS1 function thus prevents an overly narrow interpretation that confines NFS1′s role to ferroptotic regulation alone, and more accurately highlights its position as a central node within the multidimensional network of regulated cell death.

## 7. Conclusions and Future Perspectives

As an upstream core regulator of ferroptosis, NFS1 shows unique therapeutic potential in disease intervention due to its unique features in Fe-S cluster synthesis, iron homeostasis, and antioxidant defense networks. Experimental evidence suggests that inhibiting NFS1 in tumors may overcome resistance barriers by enhancing the synthetic lethality effects, while its activation or maintenance may confer potential protective benefits in highly metabolized tissues such as the myocardium. However, its double-edged sword characteristics also warn us to be extremely cautious in the pharmacological regulation to avoid systemic interventions causing mitochondrial damage and tumor tolerance. Consequently, future research urgently requires unveiling the dynamic regulation of NFS1 in different tissues, disease stages, and stress conditions. Meanwhile, the reversible time-window-dependent and dose-dependent safety margins should also be explored further. Based on the above, the combination of targeted delivery platforms, molecular degradation/activation strategies, and a multi-tiered indicator system for companion diagnostics holds the potential to advance NFS1 from fundamental research to clinical application. In summary, NFS1 not only serves as an essential lever to reveal the molecular mechanisms of ferroptosis but also holds great potential as a strategic target to achieve dual precision regulation in both tumor therapy and organ protection.

## Data Availability

Not applicable.

## Abbreviations

The following abbreviations are used in this manuscript:

AD	Alzheimer’s disease
CIA	Cytosolic iron–sulfur assembly
COXPD19	Compound oxidative phosphorylation deficiency type 19
COXPD52	Compound oxidative phosphorylation deficiency type 52
CRC	Colorectal cancer
Cys	Cysteine
DHODH	Dihydroorotate dehydrogenase
ETC	Electron transport chain
Fe-S	Iron-sulfur
GC	Gastric cancer
Glu	Glutamate
GPX4	Glutathione peroxidase 4
GSH	Glutathione
HCC	Hepatocellular carcinoma
HS	Heat stress
IMWA	Incomplete microwave ablation
IREs	Iron response elements
LC	Lung cancer
L•	Lipid radicals
LOO•	Lipid peroxy radicals
LOOH	Lipid peroxides
MWA	Microwave ablation
NFS1	Cysteine desulfurase
NO	Nitric oxide
O_2_^−^	Superoxide anion
ONOO^−^	Peroxynitrite
OXPHOS	Oxidative phosphorylation
PD	Parkinson’s disease
PUFAs	Polyunsaturated fatty acids
RCD	Regulated cell death
ROS	Reactive oxygen species
TCA	Tricarboxylic acid cycle
YAP	Yes-associated protein
•NO_2_	Nitrogen dioxide radical
•OH	Hydroxyl radicals

## References

[1] Dixon S.J. (2017). Ferroptosis: Bug or Feature?. Immunol. Rev..

[2] Hotchkiss R.S., Strasser A., McDunn J.E., Swanson P.E. (2009). Cell Death. N. Engl. J. Med..

[3] Minami J.K., Morrow D., Bayley N.A., Fernandez E.G., Salinas J.J., Tse C., Zhu H., Su B., Plawat R., Jones A. (2023). Cdkn2a Deletion Remodels Lipid Metabolism to Prime Glioblastoma for Ferroptosis. Cancer Cell.

[4] Kim J.W., Lee J.Y., Oh M., Lee E.W. (2023). An Integrated View of Lipid Metabolism in Ferroptosis Revisited via Lipidomic Analysis. Exp. Mol. Med..

[5] Zheng J., Conrad M. (2025). Ferroptosis: When Metabolism Meets Cell Death. Physiol. Rev..

[6] Li S., Zhang G., Hu J., Tian Y., Fu X. (2024). Ferroptosis at the Nexus of Metabolism and Metabolic Diseases. Theranostics.

[7] Lee J.Y., Kim W.K., Bae K.H., Lee S.C., Lee E.W. (2021). Lipid Metabolism and Ferroptosis. Biology.

[8] Tao Q., Liu N., Wu J., Chen J., Chen X., Peng C. (2024). Mefloquine Enhances the Efficacy of Anti-Pd-1 Immunotherapy via Ifn-Γ-Stat1-Irf1-Lpcat3-Induced Ferroptosis in Tumors. J. Immunother. Cancer.

[9] Reed A., Ichu T.A., Milosevich N., Melillo B., Schafroth M.A., Otsuka Y., Scampavia L., Spicer T.P., Cravatt B.F. (2022). Lpcat3 Inhibitors Remodel the Polyunsaturated Phospholipid Content of Human Cells and Protect from Ferroptosis. ACS Chem. Biol..

[10] Mou Y., Wang J., Wu J., He D., Zhang C., Duan C., Li B. (2019). Ferroptosis, a New Form of Cell Death: Opportunities and Challenges in Cancer. J. Hematol. Oncol..

[11] Friedmann Angeli J.P., Schneider M., Proneth B., Tyurina Y.Y., Tyurin V.A., Hammond V.J., Herbach N., Aichler M., Walch A., Eggenhofer E. (2014). Inactivation of the Ferroptosis Regulator Gpx4 Triggers Acute Renal Failure in Mice. Nat. Cell Biol..

[12] Tarangelo A., Magtanong L., Bieging-Rolett K.T., Li Y., Ye J., Attardi L.D., Dixon S.J. (2018). P53 Suppresses Metabolic Stress-Induced Ferroptosis in Cancer Cells. Cell Rep..

[13] Yang Y., Ma Y., Li Q., Ling Y., Zhou Y., Chu K., Xue L., Tao S. (2022). Stat6 Inhibits Ferroptosis and Alleviates Acute Lung Injury via Regulating P53/Slc7a11 Pathway. Cell Death Dis..

[14] Chen Z., Li J., Peng H., Zhang M., Wu X., Gui F., Li W., Ai F., Yu B., Liu Y. (2023). Meteorin-Like/Meteorin-B Protects Lps-Induced Acute Lung Injury by Activating Sirt1-P53-Slc7a11 Mediated Ferroptosis Pathway. Mol. Med..

[15] Jiang L., Kon N., Li T., Wang S.J., Su T., Hibshoosh H., Baer R., Gu W. (2015). Ferroptosis as a P53-Mediated Activity During Tumour Suppression. Nature.

[16] Jiang L., Hickman J.H., Wang S.J., Gu W. (2015). Dynamic Roles of P53-Mediated Metabolic Activities in Ros-Induced Stress Responses. Cell Cycle.

[17] Guo J., Zhou Y., Liu D., Wang M., Wu Y., Tang D., Liu X. (2022). Mitochondria as Multifaceted Regulators of Ferroptosis. Life Metab..

[18] Montealegre S., Lebigot E., Debruge H., Romero N., Héron B., Gaignard P., Legendre A., Imbard A., Gobin S., Lacène E. (2022). Fdx2 and Iscu Gene Variations Lead to Rhabdomyolysis with Distinct Severity and Iron Regulation. Neurol. Genet..

[19] Yang Y., Luo M., Zhang K., Zhang J., Gao T., Connell D.O., Yao F., Mu C., Cai B., Shang Y. (2020). Nedd4 Ubiquitylates Vdac2/3 to Suppress Erastin-Induced Ferroptosis in Melanoma. Nat. Commun..

[20] Wang L., Liu Y., Du T., Yang H., Lei L., Guo M., Ding H.F., Zhang J., Wang H., Chen X. (2020). Atf3 Promotes Erastin-Induced Ferroptosis by Suppressing System Xc^−^. Cell Death Differ..

[21] Chen H., Qi Q., Wu N., Wang Y., Feng Q., Jin R., Jiang L. (2022). Aspirin Promotes Rsl3-Induced Ferroptosis by Suppressing Mtor/Srebp-1/Scd1-Mediated Lipogenesis in Pik3ca-Mutant Colorectal Cancer. Redox Biol..

[22] Chen D., Xie F., Mo Y., Qin D., Zheng B., Chen L. (2025). Rsl3 Promotes Parp1 Apoptotic Functions by Distinct Mechanisms During Ferroptosis. Cell. Mol. Biol. Lett..

[23] Xie Y., Hou W., Song X., Yu Y., Huang J., Sun X., Kang R., Tang D. (2016). Ferroptosis: Process and Function. Cell Death Differ..

[24] Zilka O., Shah R., Li B., Friedmann Angeli J.P., Griesser M., Conrad M., Pratt D.A. (2017). On the Mechanism of Cytoprotection by Ferrostatin-1 and Liproxstatin-1 and the Role of Lipid Peroxidation in Ferroptotic Cell Death. ACS Cent. Sci..

[25] Zhang B., Chen X., Ru F., Gan Y., Li B., Xia W., Dai G., He Y., Chen Z. (2021). Liproxstatin-1 Attenuates Unilateral Ureteral Obstruction-Induced Renal Fibrosis by Inhibiting Renal Tubular Epithelial Cells Ferroptosis. Cell Death Dis..

[26] Miotto G., Rossetto M., Di Paolo M.L., Orian L., Venerando R., Roveri A., Vučković A.M., Bosello Travain V., Zaccarin M., Zennaro L. (2020). Insight into the Mechanism of Ferroptosis Inhibition by Ferrostatin-1. Redox Biol..

[27] Yu H., Guo P., Xie X., Wang Y., Chen G. (2017). Ferroptosis, a New Form of Cell Death, and Its Relationships with Tumourous Diseases. J. Cell. Mol. Med..

[28] Cao J.Y., Dixon S.J. (2016). Mechanisms of Ferroptosis. Cell. Mol. Life Sci..

[29] Gao M., Yi J., Zhu J., Minikes A.M., Monian P., Thompson C.B., Jiang X. (2019). Role of Mitochondria in Ferroptosis. Mol. Cell.

[30] Zhu L., Liu Z., Liu J., Li Z., Bao Y., Sun X., Zhao W., Zhou A., Wu H. (2025). Ncoa4 Linked to Endothelial Cell Ferritinophagy and Ferroptosis:A Key Regulator Aggravate Aortic Endothelial Inflammation and Atherosclerosis. Redox Biol..

[31] Mi Y., Wei C., Sun L., Liu H., Zhang J., Luo J., Yu X., He J., Ge H., Liu P. (2023). Melatonin Inhibits Ferroptosis and Delays Age-Related Cataract by Regulating Sirt6/P-Nrf2/Gpx4 and Sirt6/Ncoa4/Fth1 Pathways. Biomed. Pharmacother..

[32] Zhang Y., Liu X., Xie L., Hong J., Zhuang Q., Ren L., Li X., Zhang C. (2024). Overexpression of Nfs1 Cysteine Desulphurase Relieves Sevoflurane-Induced Neurotoxicity and Cognitive Dysfunction in Neonatal Mice via Suppressing Oxidative Stress and Ferroptosis. J. Biochem. Mol. Toxicol..

[33] Chafe S.C., Vizeacoumar F.S., Venkateswaran G., Nemirovsky O., Awrey S., Brown W.S., McDonald P.C., Carta F., Metcalfe A., Karasinska J.M. (2021). Genome-Wide Synthetic Lethal Screen Unveils Novel Caix-Nfs1/Xct Axis as a Targetable Vulnerability in Hypoxic Solid Tumors. Sci. Adv..

[34] Lin J.F., Hu P.S., Wang Y.Y., Tan Y.T., Yu K., Liao K., Wu Q.N., Li T., Meng Q., Lin J.Z. (2022). Phosphorylated Nfs1 Weakens Oxaliplatin-Based Chemosensitivity of Colorectal Cancer by Preventing Panoptosis. Signal Transduct. Target. Ther..

[35] Sewell K.E., Gola G.F., Pignataro M.F., Herrera M.G., Noguera M.E., Olmos J., Ramírez J.A., Capece L., Aran M., Santos J. (2023). Direct Cysteine Desulfurase Activity Determination by Nmr and the Study of the Functional Role of Key Structural Elements of Human Nfs1. ACS Chem. Biol..

[36] Alvarez S.W., Sviderskiy V.O., Terzi E.M., Papagiannakopoulos T., Moreira A.L., Adams S., Sabatini D.M., Birsoy K., Possemato R. (2017). Nfs1 Undergoes Positive Selection in Lung Tumours and Protects Cells from Ferroptosis. Nature.

[37] Jiang Y., Li L., Li W., Liu K., Wu Y., Wang Z. (2024). Nfs1 Inhibits Ferroptosis in Gastric Cancer by Regulating the *Stat3* Pathway. J. Bioenerg. Biomembr..

[38] Huang J., Xie H., Li J., Huang X., Cai Y., Yang R., Yang D., Bao W., Zhou Y., Li T. (2025). Histone Lactylation Drives Liver Cancer Metastasis by Facilitating Nsf1-Mediated Ferroptosis Resistance after Microwave Ablation. Redox Biol..

[39] She G., Hai X.X., Jia L.Y., Zhang Y.J., Ren Y.J., Pang Z.D., Wu L.H., Han M.Z., Zhang Y., Li J.J. (2025). Hippo Pathway Activation Mediates Cardiomyocyte Ferroptosis to Promote Dilated Cardiomyopathy Through Downregulating Nfs1. Redox Biol..

[40] Wang M., Zhang S., Tian J., Yang F., Chen H., Bai S., Kang J., Pang K., Huang J., Dong M. (2025). Impaired Iron-Sulfur Cluster Synthesis Induces Mitochondrial Parthanatos in Diabetic Cardiomyopathy. Adv. Sci..

[41] Wang Y., Ying X., Wang Y., Zou Z., Yuan A., Xiao Z., Geng N., Qiao Z., Li W., Lu X. (2023). Hydrogen Sulfide Alleviates Mitochondrial Damage and Ferroptosis by Regulating Opa3-Nfs1 Axis in Doxorubicin-Induced Cardiotoxicity. Cell Signal.

[42] Ru Q., Li Y., Chen L., Wu Y., Min J., Wang F. (2024). Iron Homeostasis and Ferroptosis in Human Diseases: Mechanisms and Therapeutic Prospects. Signal Transduct. Target. Ther..

[43] Sun S., Shen J., Jiang J., Wang F., Min J. (2023). Targeting Ferroptosis Opens New Avenues for the Development of Novel Therapeutics. Signal Transduct. Target. Ther..

[44] Fukumoto J., Lin M., Banday M.M., Patil S.S., Krishnamurthy S., Breitzig M., Soundararajan R., Galam L., Narala V.R., Johns C. (2022). Aberrant Expression of Aco1 in Vasculatures Parallels Progression of Idiopathic Pulmonary Fibrosis. Front. Pharmacol..

[45] Read A.D., Bentley R.E., Archer S.L., Dunham-Snary K.J. (2021). Mitochondrial Iron-Sulfur Clusters: Structure, Function, and an Emerging Role in Vascular Biology. Redox Biol..

[46] Steinhilper R., Boß L., Freibert S.A., Schulz V., Krapoth N., Kaltwasser S., Lill R., Murphy B.J. (2024). Two-Stage Binding of Mitochondrial Ferredoxin-2 to the Core Iron-Sulfur Cluster Assembly Complex. Nat. Commun..

[47] Schulz V., Steinhilper R., Oltmanns J., Freibert S.A., Krapoth N., Linne U., Welsch S., Hoock M.H., Schünemann V., Murphy B.J. (2024). Mechanism and Structural Dynamics of Sulfur Transfer During De Novo [2fe-2s] Cluster Assembly on Iscu2. Nat. Commun..

[48] Mühlenhoff U., Balk J., Richhardt N., Kaiser J.T., Sipos K., Kispal G., Lill R. (2004). Functional Characterization of the Eukaryotic Cysteine Desulfurase Nfs1p from Saccharomyces Cerevisiae. J. Biol. Chem..

[49] Nakai Y., Nakai M., Hayashi H., Kagamiyama H. (2001). Nuclear Localization of Yeast Nfs1p Is Required for Cell Survival. J. Biol. Chem..

[50] Kovárová J., Horáková E., Changmai P., Vancová M., Lukeš J. (2014). Mitochondrial and Nucleolar Localization of Cysteine Desulfurase Nfs and the Scaffold Protein Isu in Trypanosoma Brucei. Eukaryot. Cell.

[51] Chowdhury M.M., Dosche C., Löhmannsröben H.G., Leimkühler S. (2012). Dual Role of the Molybdenum Cofactor Biosynthesis Protein Mocs3 in Trna Thiolation and Molybdenum Cofactor Biosynthesis in Humans. J. Biol. Chem..

[52] Marelja Z., Stöcklein W., Nimtz M., Leimkühler S. (2008). A Novel Role for Human Nfs1 in the Cytoplasm: Nfs1 Acts as a Sulfur Donor for Mocs3, a Protein Involved in Molybdenum Cofactor Biosynthesis. J. Biol. Chem..

[53] Paul V.D., Lill R. (2015). Biogenesis of Cytosolic and Nuclear Iron-Sulfur Proteins and Their Role in Genome Stability. Biochim. Biophys. Acta.

[54] Kim J.Y., Kim J.K., Kim H. (2020). Abcb7 Simultaneously Regulates Apoptotic and Non-Apoptotic Cell Death by Modulating Mitochondrial Ros and Hif1α-Driven Nfκb Signaling. Oncogene.

[55] Maclean A.E., Sloan M.A., Renaud E.A., Argyle B.E., Lewis W.H., Ovciarikova J., Demolombe V., Waller R.F., Besteiro S., Sheiner L. (2024). The Toxoplasma Gondii Mitochondrial Transporter Abcb7l Is Essential for the Biogenesis of Cytosolic and Nuclear Iron-Sulfur Cluster Proteins and Cytosolic Translation. mBio.

[56] Netz D.J., Stith C.M., Stümpfig M., Köpf G., Vogel D., Genau H.M., Stodola J.L., Lill R., Burgers P.M., Pierik A.J. (2011). Eukaryotic DNA Polymerases Require an Iron-Sulfur Cluster for the Formation of Active Complexes. Nat. Chem. Biol..

[57] Ter Beek J., Parkash V., Bylund G.O., Osterman P., Sauer-Eriksson A.E., Johansson E. (2019). Structural Evidence for an Essential Fe-S Cluster in the Catalytic Core Domain of DNA Polymerase ϵ. Nucleic Acids Res..

[58] Guan X., Yang Z., Wang J., Lu W., Wang S., Yang M., Sun P., Hu W., Yang L., Li H. (2025). Naringin Attenuates Myocardial Ischemia-Reperfusion Injury by Promoting Mitochondrial Translocation of Ndufs1 and Suppressing Cardiac Microvascular Endothelial Cell Ferroptosis. J. Nutr. Biochem..

[59] Schulz V., Basu S., Freibert S.A., Webert H., Boss L., Mühlenhoff U., Pierrel F., Essen L.O., Warui D.M., Booker S.J. (2023). Functional Spectrum and Specificity of Mitochondrial Ferredoxins Fdx1 and Fdx2. Nat. Chem. Biol..

[60] Sheftel A.D., Stehling O., Pierik A.J., Elsässer H.P., Mühlenhoff U., Webert H., Hobler A., Hannemann F., Bernhardt R., Lill R. (2010). Humans Possess Two Mitochondrial Ferredoxins, Fdx1 and Fdx2, with Distinct Roles in Steroidogenesis, Heme, and Fe/S Cluster Biosynthesis. Proc. Natl. Acad. Sci. USA.

[61] Chen S., Chen T., Xu C., Yu X., Shi J., Yang C., Zhu T. (2025). Iron Overload Exaggerates Renal Ischemia-Reperfusion Injury by Promoting Tubular Cuproptosis via Interrupting Function of Lias. Redox Biol..

[62] Hendricks A.L., Wachnowsky C., Fries B., Fidai I., Cowan J.A. (2021). Characterization and Reconstitution of Human Lipoyl Synthase (Lias) Supports Isca2 and Iscu as Primary Cluster Donors and an Ordered Mechanism of Cluster Assembly. Int. J. Mol. Sci..

[63] Liang D., Minikes A.M., Jiang X. (2022). Ferroptosis at the Intersection of Lipid Metabolism and Cellular Signaling. Mol. Cell.

[64] Fujihara K.M., Zhang B.Z., Jackson T.D., Ogunkola M.O., Nijagal B., Milne J.V., Sallman D.A., Ang C.S., Nikolic I., Kearney C.J. (2022). Eprenetapopt Triggers Ferroptosis, Inhibits Nfs1 Cysteine Desulfurase, and Synergizes with Serine and Glycine Dietary Restriction. Sci. Adv..

[65] Zhu Z., Gan H., Wang Y., Jia G., Li H., Ma Z., Wang J., Shang X., Niu W. (2025). Identification of a Selective Inhibitor of Human Nfs1, a Cysteine Desulfurase Involved in Fe-S Cluster Assembly, via Structure-Based Virtual Screening. Int. J. Mol. Sci..

[66] Ding X., Cui L., Mi Y., Hu J., Cai Z., Tang Q., Yang L., Yang Z., Wang Q., Li H. (2025). Ferroptosis in Cancer: Revealing the Multifaceted Functions of Mitochondria. Cell. Mol. Life Sci..

[67] Sviderskiy V.O., Blumenberg L., Gorodetsky E., Karakousi T.R., Hirsh N., Alvarez S.W., Terzi E.M., Kaparos E., Whiten G.C., Ssebyala S. (2020). Hyperactive Cdk2 Activity in Basal-Like Breast Cancer Imposes a Genome Integrity Liability That Can Be Exploited by Targeting DNA Polymerase E. Mol. Cell.

[68] Murray J., Taylor S.W., Zhang B., Ghosh S.S., Capaldi R.A. (2003). Oxidative Damage to Mitochondrial Complex I Due to Peroxynitrite: Identification of Reactive Tyrosines by Mass Spectrometry. J. Biol. Chem..

[69] Dewan A., Jain C., Das M., Tripathi A., Sharma A.K., Singh H., Malhotra N., Seshasayee A.S.N., Chakrapani H., Singh A. (2024). Intracellular Peroxynitrite Perturbs Redox Balance, Bioenergetics, and Fe-S Cluster Homeostasis in Mycobacterium Tuberculosis. Redox Biol..

[70] Maiti A.K., Spoorthi B.C., Saha N.C., Panigrahi A.K. (2018). Mitigating Peroxynitrite Mediated Mitochondrial Dysfunction in Aged Rat Brain by Mitochondria-Targeted Antioxidant Mitoq. Biogerontology.

[71] Sun D., Wang L., Wu Y., Yu Y., Yao Y., Yang H., Hao C. (2025). Lipid Metabolism in Ferroptosis: Mechanistic Insights and Therapeutic Potential. Front. Immunol..

[72] Rochette L., Dogon G., Rigal E., Zeller M., Cottin Y., Vergely C. (2022). Lipid Peroxidation and Iron Metabolism: Two Corner Stones in the Homeostasis Control of Ferroptosis. Int. J. Mol. Sci..

[73] Radi R., Beckman J.S., Bush K.M., Freeman B.A. (1991). Peroxynitrite-Induced Membrane Lipid Peroxidation: The Cytotoxic Potential of Superoxide and Nitric Oxide. Arch. Biochem. Biophys..

[74] Pérez de la Lastra J.M., Juan C.A., Plou F.J., Pérez-Lebeña E. (2022). The Nitration of Proteins, Lipids and DNA by Peroxynitrite Derivatives-Chemistry Involved and Biological Relevance. Stresses.

[75] Manivarma T., Kapralov A.A., Samovich S.N., Tyurina Y.Y., Tyurin V.A., VanDemark A.P., Nowak W., Bayır H., Bahar I., Kagan V.E. (2023). Membrane Regulation of 15lox-1/Pebp1 Complex Prompts the Generation of Ferroptotic Signals, Oxygenated Pes. Free Radic. Biol. Med..

[76] Pei J., Pan X., Wei G., Hua Y. (2023). Research Progress of Glutathione Peroxidase Family (Gpx) in Redoxidation. Front. Pharmacol..

[77] Zhou L., Lian G., Zhou T., Cai Z., Yang S., Li W., Cheng L., Ye Y., He M., Lu J. (2025). Palmitoylation of Gpx4 via the Targetable Zdhhc8 Determines Ferroptosis Sensitivity and Antitumor Immunity. Nat. Cancer.

[78] Huang B., Wang H., Liu S., Hao M., Luo D., Zhou Y., Huang Y., Nian Y., Zhang L., Chu B. (2025). PPalmitoylation-Dependent Regulation of Gpx4 Suppresses Ferroptosis. Nat. Commun..

[79] Koppula P., Zhang Y., Zhuang L., Gan B. (2018). Amino Acid Transporter Slc7a11/Xct at the Crossroads of Regulating Redox Homeostasis and Nutrient Dependency of Cancer. Cancer Commun..

[80] Chen Q., Zheng W., Guan J., Liu H., Dan Y., Zhu L., Song Y., Zhou Y., Zhao X., Zhang Y. (2023). Socs2-Enhanced Ubiquitination of Slc7a11 Promotes Ferroptosis and Radiosensitization in Hepatocellular Carcinoma. Cell Death Differ..

[81] Liu J., Gao W., Sheng Y., Sun J., Wen D. (2023). Resveratrol Drives Ferroptosis of Acute Myeloid Leukemia Cells Through Hsa-Mir-335-5p/Nfs1/Gpx4 Pathway in a Ros-Dependent Manner. Cell. Mol. Biol..

[82] Li R., Yuan H., Zhang C., Han D., Wang Y., Feng L. (2024). Induced Ferroptosis Pathway by Regulating Cellular Lipid Peroxidation with Peroxynitrite Generator for Reversing “Cold” Tumors. Small.

[83] Chen Y., Huang X., Hu R., Lu E., Luo K., Yan X., Zhang Z., Ma Y., Zhang M., Sha X. (2025). Inhalable Biomimetic Polyunsaturated Fatty Acid-Based Nanoreactors for Peroxynitrite-Augmented Ferroptosis Potentiate Radiotherapy in Lung Cancer. J. Nanobiotechnol..

[84] Stockwell B.R. (2022). Ferroptosis Turns 10: Emerging Mechanisms, Physiological Functions, and Therapeutic Applications. Cell.

[85] Chen D., Chu B., Yang X., Liu Z., Jin Y., Kon N., Rabadan R., Jiang X., Stockwell B.R., Gu W. (2021). Ipla2β-Mediated Lipid Detoxification Controls P53-Driven Ferroptosis Independent of Gpx4. Nat. Commun..

[86] Wang C.K., Chen T.J., Tan G.Y.T., Chang F.P., Sridharan S., Yu C.A., Chang Y.H., Chen Y.J., Cheng L.T., Hwang-Verslues W.W. (2023). Mex3a Mediates P53 Degradation to Suppress Ferroptosis and Facilitate Ovarian Cancer Tumorigenesis. Cancer Res..

[87] Liu Y., Gu W. (2022). P53 in Ferroptosis Regulation: The New Weapon for the Old Guardian. Cell Death Differ..

[88] Xu R., Wang W., Zhang W. (2023). Ferroptosis and the Bidirectional Regulatory Factor P53. Cell Death Discov..

[89] Jiang Y., Li W., Zhang J., Liu K., Wu Y., Wang Z. (2024). Nfs1 as a Candidate Prognostic Biomarker for Gastric Cancer Correlated with Immune Infiltrates. Int. J. Gen. Med..

[90] Shi X., Zhao Y., Gao H.Y., Yang W., Liao J., Wang H.H., Wang X.T., Yan W. (2025). Nfs1, Together with Fxn, Protects Cells from Ferroptosis and DNA Damage in Diffuse Large B-Cell Lymphoma. Redox Biol..

[91] Hershkovitz T., Kurolap A., Tal G., Paperna T., Mory A., Staples J., Brigatti K.W., Gonzaga-Jauregui C., Dumin E., Saada A. (2021). A Recurring Nfs1 Pathogenic Variant Causes a Mitochondrial Disorder with Variable Intra-Familial Patient Outcomes. Mol. Genet. Metab. Rep..

[92] Yang J.H., Friederich M.W., Ellsworth K.A., Frederick A., Foreman E., Malicki D., Dimmock D., Lenberg J., Prasad C., Yu A.C. (2022). Expanding the Phenotypic and Molecular Spectrum of Nfs1-Related Disorders That Cause Functional Deficiencies in Mitochondrial and Cytosolic Iron-Sulfur Cluster Containing Enzymes. Hum. Mutat..

[93] Farhan S.M., Wang J., Robinson J.F., Lahiry P., Siu V.M., Prasad C., Kronick J.B., Ramsay D.A., Rupar C.A., Hegele R.A. (2014). Exome Sequencing Identifies Nfs1 Deficiency in a Novel Fe-S Cluster Disease, Infantile Mitochondrial Complex Ii/Iii Deficiency. Mol. Genet. Genomic Med..

[94] Lim S.C., Friemel M., Marum J.E., Tucker E.J., Bruno D.L., Riley L.G., Christodoulou J., Kirk E.P., Boneh A., DeGennaro C.M. (2013). Mutations in Lyrm4, Encoding Iron-Sulfur Cluster Biogenesis Factor Isd11, Cause Deficiency of Multiple Respiratory Chain Complexes. Hum. Mol. Genet..

[95] Saha P.P., Srivastava S., Kumar S.K.P., Sinha D., D’Silva P. (2015). Mapping Key Residues of Isd11 Critical for Nfs1-Isd11 Subcomplex Stability: Implications in the Development of Mitochondrial Disorder, Coxpd19. J. Biol. Chem..

[96] Adam A.C., Bornhövd C., Prokisch H., Neupert W., Hell K. (2006). The Nfs1 Interacting Protein Isd11 Has an Essential Role in Fe/S Cluster Biogenesis in Mitochondria. EMBO J..

[97] Li Y., Liu C., Fang B., Chen X., Wang K., Xin H., Wang K., Yang S.M. (2024). Ferroptosis, a Therapeutic Target for Cardiovascular Diseases, Neurodegenerative Diseases and Cancer. J. Transl. Med..

[98] Alves F., Lane D., Nguyen T.P.M., Bush A.I., Ayton S. (2025). In Defence of Ferroptosis. Signal Transduct. Target. Ther..

[99] Wen R.J., Dong X., Zhuang H.W., Pang F.X., Ding S.C., Li N., Mai Y.X., Zhou S.T., Wang J.Y., Zhang J.F. (2023). Baicalin Induces Ferroptosis in Osteosarcomas through a Novel Nrf2/Xct/Gpx4 Regulatory Axis. Phytomedicine.

[100] Liu Y., Mi Y., Wang Y., Meng Q., Xu L., Liu Y., Zhou D., Wang Y., Liang D., Li W. (2023). Loureirin C Inhibits Ferroptosis After Cerebral Ischemia Reperfusion Through Regulation of the Nrf2 Pathway in Mice. Phytomedicine.

[101] Xu S., Wu B., Zhong B., Lin L., Ding Y., Jin X., Huang Z., Lin M., Wu H., Xu D. (2021). Naringenin Alleviates Myocardial Ischemia/Reperfusion Injury by Regulating the Nuclear Factor-Erythroid Factor 2-Related Factor 2 (Nrf2) /System Xc^−^/ Glutathione Peroxidase 4 (Gpx4) Axis to Inhibit Ferroptosis. Bioengineered.

[102] Magtanong L., Ko P.J., To M., Cao J.Y., Forcina G.C., Tarangelo A., Ward C.C., Cho K., Patti G.J., Nomura D.K. (2019). Exogenous Monounsaturated Fatty Acids Promote a Ferroptosis-Resistant Cell State. Cell Chem. Biol..

[103] Koppula P., Zhuang L., Gan B. (2021). Cystine Transporter Slc7a11/Xct in Cancer: Ferroptosis, Nutrient Dependency, and Cancer Therapy. Protein Cell.

[104] Uzarska M.A., Grochowina I., Soldek J., Jelen M., Schilke B., Marszalek J., Craig E.A., Dutkiewicz R. (2022). During Fes Cluster Biogenesis, Ferredoxin and Frataxin Use Overlapping Binding Sites on Yeast Cysteine Desulfurase Nfs1. J. Biol. Chem..

[105] Zangari J., Stehling O., Freibert S.A., Bhattacharya K., Rouaud F., Serre-Beinier V., Maundrell K., Montessuit S., Ferre S.M., Vartholomaiou E. (2025). D-Cysteine Impairs Tumour Growth by Inhibiting Cysteine Desulfurase Nfs1. Nat. Metab..

[106] Mao Z., Shi Z., Cui M., Ma X., Wang Y., Zhang X., Jing R., Wang J. (2025). Overexpression of the Ferroptosis-Related Gene, Nfs1, Corresponds to Gastric Cancer Growth and Tumor Immune Infiltration. Open Life Sci..

[107] Pang Y., Tan G., Yang X., Lin Y., Chen Y., Zhang J., Xie T., Zhou H., Fang J., Zhao Q. (2021). Iron-Sulphur Cluster Biogenesis Factor Lyrm4 Is a Novel Prognostic Biomarker Associated with Immune Infiltrates in Hepatocellular Carcinoma. Cancer Cell Int..

[108] Hu Q., Wei W., Wu D., Huang F., Li M., Li W., Yin J., Peng Y., Lu Y., Zhao Q. (2022). Blockade of Gch1/Bh4 Axis Activates Ferritinophagy to Mitigate the Resistance of Colorectal Cancer to Erastin-Induced Ferroptosis. Front. Cell Dev. Biol..

[109] Ghosh M.C., Zhang D.L., Jeong S.Y., Kovtunovych G., Ollivierre-Wilson H., Noguchi A., Tu T., Senecal T., Robinson G., Crooks D.R. (2013). Deletion of Iron Regulatory Protein 1 Causes Polycythemia and Pulmonary Hypertension in Mice Through Translational Derepression of Hif2α. Cell Metab..

[110] Guo J., Xu B., Han Q., Zhou H., Xia Y., Gong C., Dai X., Li Z., Wu G. (2018). Ferroptosis: A Novel Anti-Tumor Action for Cisplatin. Cancer Res. Treat..

[111] Zhang X., Sui S., Wang L., Li H., Zhang L., Xu S., Zheng X. (2020). Inhibition of Tumor Propellant Glutathione Peroxidase 4 Induces Ferroptosis in Cancer Cells and Enhances Anticancer Effect of Cisplatin. J. Cell. Physiol..

[112] Lei G., Zhang Y., Koppula P., Liu X., Zhang J., Lin S.H., Ajani J.A., Xiao Q., Liao Z., Wang H. (2020). The Role of Ferroptosis in Ionizing Radiation-Induced Cell Death and Tumor Suppression. Cell Res..

[113] Springer A.D., Dowdy S.F. (2018). Galnac-Sirna Conjugates: Leading the Way for Delivery of Rnai Therapeutics. Nucleic Acid. Ther..

[114] Chamberlain K., Riyad J.M., Weber T. (2017). Cardiac Gene Therapy with Adeno-Associated Virus-Based Vectors. Curr. Opin. Cardiol..

[115] Inagaki K., Fuess S., Storm T.A., Gibson G.A., McTiernan C.F., Kay M.A., Nakai H. (2006). Robust Systemic Transduction with Aav9 Vectors in Mice: Efficient Global Cardiac Gene Transfer Superior to That of Aav8. Mol. Ther..

[116] Shu L., Luo P., Chen Q., Liu J., Huang Y., Wu C., Pan X., Huang Z. (2024). Fibroin Nanodisruptor with Ferroptosis-Autophagy Synergism Is Potent for Lung Cancer Treatment. Int. J. Pharm..

[117] Maeda H., Nakamura H., Fang J. (2013). The Epr Effect for Macromolecular Drug Delivery to Solid Tumors: Improvement of Tumor Uptake, Lowering of Systemic Toxicity, and Distinct Tumor Imaging in Vivo. Adv. Drug Deliv. Rev..

[118] Yan S., Na J., Liu X., Wu P. (2024). Different Targeting Ligands-Mediated Drug Delivery Systems for Tumor Therapy. Pharmaceutics.

[119] Debacker A.J., Voutila J., Catley M., Blakey D., Habib N. (2020). Delivery of Oligonucleotides to the Liver with Galnac: From Research to Registered Therapeutic Drug. Mol. Ther..

[120] Grommes C., Oxnard G.R., Kris M.G., Miller V.A., Pao W., Holodny A.I., Clarke J.L., Lassman A.B. (2011). “Pulsatile” High-Dose Weekly Erlotinib for Cns Metastases from Egfr Mutant Non-Small Cell Lung Cancer. Neuro Oncol..

[121] Tong W.H., Rouault T.A. (2024). In-Gel Activity Assay of Mammalian Mitochondrial and Cytosolic Aconitases, Surrogate Markers of Compartment-Specific Oxidative Stress and Iron Status. Bio-Protoc..

[122] Dixon S.J., Lemberg K.M., Lamprecht M.R., Skouta R., Zaitsev E.M., Gleason C.E., Patel D.N., Bauer A.J., Cantley A.M., Yang W.S. (2012). Ferroptosis: An Iron-Dependent Form of Nonapoptotic Cell Death. Cell.

[123] Xia F., George S.L., Ning J., Li L., Huang X. (2021). A Signature Enrichment Design with Bayesian Adaptive Randomization. J. Appl. Stat..

[124] Wang H., Yee D. (2019). I-Spy 2: A Neoadjuvant Adaptive Clinical Trial Designed to Improve Outcomes in High-Risk Breast Cancer. Curr. Breast Cancer Rep..

[125] Li Y., Wang X. (2025). A Metabolic Perspective on Cuproptosis. Trends Endocrinol. Metab..

[126] Tralongo P., Ballato M., Fiorentino V., Giordano W.G., Zuccalà V., Pizzimenti C., Bakacs A., Ieni A., Tuccari G., Fadda G. (2025). Cuproptosis: A Review on Mechanisms, Role in Solid and Hematological Tumors, and Association with Viral Infections. Mediterr. J. Hematol. Infect. Dis..

[127] Guo Z., Chen D., Yao L., Sun Y., Li D., Le J., Dian Y., Zeng F., Chen X., Deng G. (2025). The Molecular Mechanism and Therapeutic Landscape of Copper and Cuproptosis in Cancer. Signal Transduct. Target. Ther..

